# Differential wedging of vertebral body and intervertebral disc in thoracic and lumbar spine in adolescent idiopathic scoliosis – A cross sectional study in 150 patients

**DOI:** 10.1186/1748-7161-3-11

**Published:** 2008-08-13

**Authors:** Hitesh N Modi, Seung Woo Suh, Hae-Ryong Song, Jae-Hyuk Yang, Hak-Jun Kim, Chetna H Modi

**Affiliations:** 1 Scolioisis Research Institute, Dept of Orthopaedics, Korea University Guro Hospital, Seoul, Korea; 2 Rare Disease Institute, Dept of Orthopaedics, Korea University Guro Hospital, Seoul, Korea; 3 Veterans Severance Hospital, Dept of Orthopaedics, Seoul, Korea

## Abstract

**Background:**

Hueter-Volkmann's law regarding growth modulation suggests that increased pressure on the end plate of bone retards the growth (Hueter) and conversely, reduced pressure accelerates the growth (Volkmann). Literature described the same principle in Rat-tail model. Human spine and its deformity i.e. scoliosis has also same kind of pattern during the growth period which causes wedging in disc or vertebral body.

**Methods:**

This cross sectional study in 150 patients of adolescent idiopathic scoliosis was done to evaluate vertebral body and disc wedging in scoliosis and to compare the extent of differential wedging of body and disc, in thoracic and lumbar area. We measured wedging of vertebral bodies and discs, along with two adjacent vertebrae and disc, above and below the apex and evaluated them according to severity of curve (curve < 30° and curve > 30°) to find the relationship of vertebral body or disc wedging with scoliosis in thoracic and lumbar spine. We also compared the wedging and rotations of vertebrae.

**Results:**

In both thoracic and lumbar curves, we found that greater the degree of scoliosis, greater the wedging in both disc and body and the degree of wedging was more at apex supporting the theory of growth retardation in stress concentration area. However, the degree of wedging in vertebral body is more than the disc in thoracic spine while the wedging was more in disc than body in lumbar spine. On comparing the wedging with the rotation, we did not find any significant relationship suggesting that it has no relation with rotation.

**Conclusion:**

From our study, we can conclude that wedging in disc and body are increasing with progression on scoliosis and maximum at apex; however there is differential wedging of body and disc, in thoracic and lumbar area, that is vertebral body wedging is more profound in thoracic area while disc wedging is more profound in lumbar area which possibly form 'vicious cycle' by asymmetric loading to spine for the progression of curve.

## Background

Scoliosis is a three-dimensional deformity involving coronal, sagittal and axial angulations. Extent of the disease progress, decision to change the treatment strategy and efficacy of the treatment are mainly dependent on the severity in coronal plane angle, commonly known as Cobb's angle as a primary interest. In idiopathic scoliosis, factors responsible for the progression of curve are still unclear and authors have their own point of view in the literature. It has been suggested by Taylor et al. [1] that the deformity begins in the vertebral disc and then the vertebrae become more deformed as the scoliosis progresses while others proposed that vertebral wedging is the most essential factor in scoliosis progression [2,3]. Perdriolle et al [3] showed that wedging is not only the most essential deformation, but it has also a direct correlation with Cobb's angle. Xiong et al [4], found that wedging starts very early in scoliosis and it is a simultaneous wedging of body and disc. Thus, whether there is an intrinsic disturbance in the vertebral body or the disc or whether it is the extra osseous factors leading to scoliosis, is still unknown. The progression of the spinal deformities is due to growth modulation either in body or disc or both in relation with stress concentration effect by the Hueter-Volkmann law [5]. The Hueter-Volkmann's law can be explained as follows: In the skeletally Immature, bone growth is retarded in areas of increased pressure (Hueter) and relatively reversed when pressure is withdrawn (Volkmann). Many studies have authenticated this law on animals [6,2-12], in which localized forces were applied to produce compression and distraction over short segments of spine but none of them have tried to analyze its effect on the spine as progression of disease. Roaf [13] has proposed a vicious cycle for progression of kyphosis. Based on this law, we have commonly observed that proportion of vertebral wedging in lumbar spine is minimal even in acute curves when it is compared with thoracic spine. Recently Stokes [14,15] has supported the 'vicious cycle' theory of scoliosis progression that proposes that scoliosis causes asymmetrical spinal loading and consequentially asymmetrical spinal growth. Stokes and Aronsson [11] have shown that in idiopathic or neuromuscular scoliosis, disc wedging is higher in thoracic region and vertebral body wedging is higher in lumbar or thoraco-lumbar level; and suggested importance of anatomic region for the proportion of wedging. We measured wedging of vertebra and disc in thoracic and lumbar spine in a large sample with adolescent idiopathic scoliosis, and compared them to know whether the wedging, if found, was significant. This indicates that if wedging increases with increase curve, it causes stress concentration and growth modulation causing the progression of curve. Thus the main goals of this paper are 1) to study the difference in the wedging of disc and vertebral body between thoracic and lumbar spine, and 2) to compare the amount of wedging according to severity of the scoliosis curve.

## Methods

**Figure 1:**
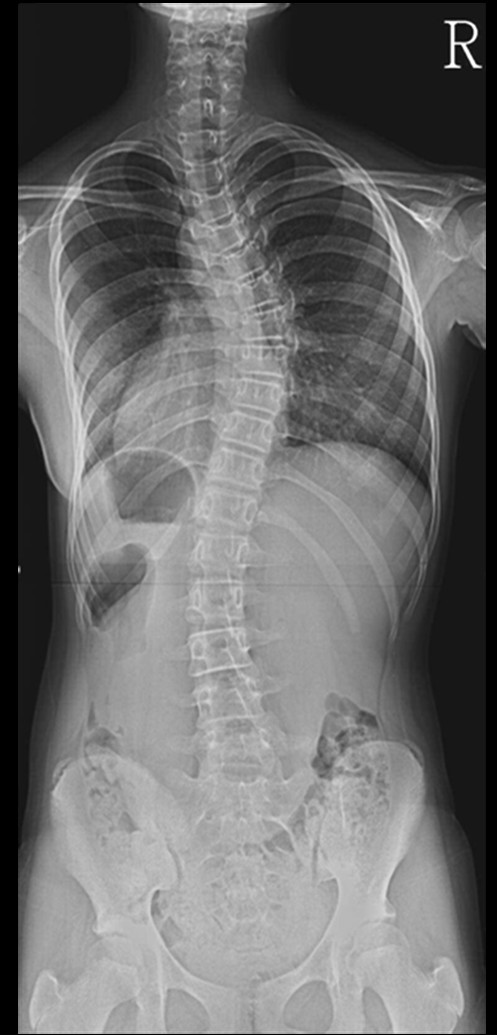
Roentgen graphic assessment was performed with whole spine standing radiogram including pelvis in all patients.

150 patients with adolescent idiopathic scoliosis were studied in this cross sectional analysis. Their Cobb's angle was 10°~60° in 141 thoracic and 120 lumbar curves from 21 thoracic, 4 lumbar and 116 with thoracic and lumbar curves, in 122 females and 28 males with their average age 14.2 (range, 11~20) years. All patients underwent for radiogram in form of antero-posterior view of whole spine (figure 1) in standing position with the use of 500 mA standard radiography machine keeping 72 inches distance from body to the tube as standard. We excluded those patients who had kyphoscoliosis or lordoscoliosis from our study because these patients also showed thoracic hyperkyphosis > 60° or lumbar hyperlordosis > 60° on lateral view of spinal radiogram. The reason for excluding these patients that hyperkyphosis or hyperlordosis will not produce only lateral wedging but they will also produce antero-posterior wedging also which we did not want to include. We divided the patient in two groups according to the severity of curves, curve less than 30° and curve more than 30°. We also evaluated patients according to the thoracic and lumbar curve patterns and therefore, the patients' curve, having thoracic and lumbar curves were considered as two separate curve patterns. We also excluded thoracolumbar and double thoracic curves from our study. Thus, there were 21 thoracic and 4 lumbar single curves and 116 patients with double (thoracic and lumbar) curves. All thoracic curves including those from double curves were grouped together in thoracic and similarly, all lumbar curves were grouped in lumbar curves. Therefore, there were 141 thoracic and 120 lumbar curves.

**Figure 2:**
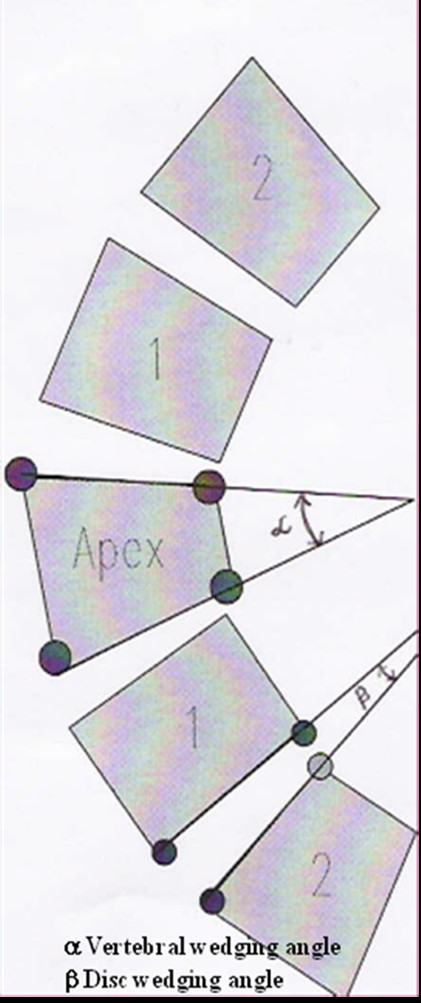
**Measurement of vertebral body and disc wedging. 1) **The vertebral wedge (α) angle was measured with angle formed by two lines connecting upper endplate and lower endplate of vertebra. **2) **The angle between the inferior end plate of the upper vertebra and superior end plate of the lower vertebra was measured to calculate the intervertebral disc wedge (β) angle.

The average Cobb's angle was 27.11° (range, 10°~60°) for thoracic and 25.13° (range, 10°~60°) for lumbar curves. Vertebral body and disc wedging (figure 2) were measured using Inner space 2-D computer software. We measured wedging of the apical vertebra along with two adjacent vertebrae, above and below the apex, and wedging of the end vertebrae of curves. Those curves, which had apex at intervertebral disc, the upper vertebral body was considered as the apical vertebra. Similarly, five discs around the apex of the curve, wedging for the discs were measured, including the apical disc and two discs above and below the apex. In those, where the apex was at the vertebral level the lower disc was selected as the apical disc. The vertebral wedge angle measurement was done by drawing a line between the superior and inferior end plates of each vertebra in the curve. Similarly, the angle between the inferior end plate of the upper vertebra and superior end plate of the lower vertebra were measured to calculate the intervertebral disc-wedge angle. Two spine fellows measured all angles independently and then average value were taken for calculation to reduce inter-observer errors (r = 0.96 for vertebral bodies and r = 0.97 for discs; Pearson correlation coefficient).

We compared results of the vertebral body wedging of each curve with the corresponding disc wedging of the same region to ascertain whether there was any significance between two. In addition, the wedging in the apical vertebra was compared with the other vertebrae above and below it. Similarly, we also compared wedging at the apical disc with the other discs above and below. The vertebral rotation of each vertebra was graded as per Nash and Moe [16], which produced ordinal data not the continuous data; and we compared vertebral wedging with different grades of vertebral rotation.

### Statistical method

Vertebral body wedging and disc wedging in both thoracic and lumbar spine were compared with paired t-test. We hypothesized that maximum wedging would be at the apical disc or body when compared with the adjacent levels and, as severity of Cobb's angle increased, wedging at the apical disc or body would also increase. Therefore, in thoracic spine, comparison among the wedging of the apical vertebra with four vertebrae above and below was done using the ANOVA (Analysis of Variance) test. Unpaired t-test was used to compare the apical thoracic body wedging between curves less than 30° and curves more than 30°. In lumbar spine, comparison of the apical disc wedging with the wedging of four discs, above and below the apex, was done using ANOVA test and similarly, the apical lumbar discs wedging were compared according to severity of curves using unpaired t-test. Additionally we have used analysis of covariance to find out relationship between Cobb angle and wedging in the apical disc and body. For all these statistical methods, 'P' value less than 0.05 was considered significant.

## Results

**Figure 3:**
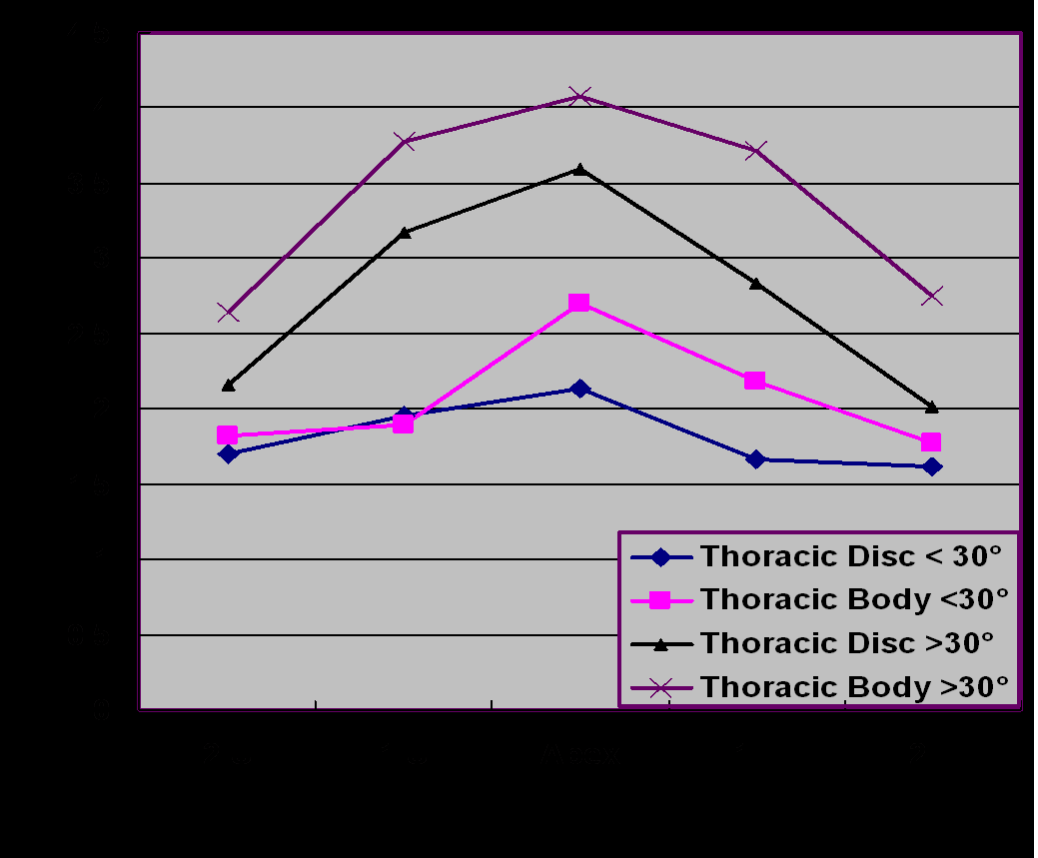
**Wedging in body and disc in thoracic spine**. In thoracic spine, the vertebral wedging is significantly greater than the disc wedging. **X-axis **shows level of wedging measurement. 1 U: 1st upper body or disc. 2 U: 2nd upper body or disc. 1 L: 1st lower body or disc. 2 L: 2nd lower or disc. **Y-axis **shows wedging in of body or disc in degree.

Our results indicate that in thoracic spine, vertebral body wedging (mean 2.7° with curves less than 30° and mean 4.1° with greater than 30°) in the apical vertebrae is significantly greater than the disc wedging (p = 0.0097, paired t-test) (Figure 3). The vertebral body wedging was mainly towards the concavity of the curve. In addition, vertebral body wedging was not confined to the apex alone, but end vertebrae also demonstrated wedging. The wedging was most profound in the apical vertebra having an average wedging of 3.17° and it gradually decreased towards the end vertebra having an average of 1.99° and 1.58° at upper and lower end respectively. The apical vertebral wedging was found to be significantly more than the vertebral wedging above and below (p < 0.0001, ANOVA test). Even the disc wedging in the thoracic spine was profound at the apex having an average of 2.63° compared to the disc level above and below (p < 0.0001, ANOVA test) (table 1).

**Table 1:** Average wedging of thoracic and lumbar spine

**AREA**	**No. of patients**	**Wedging Angle in Degrees ± SD**				
		**2U**	**1U**	**APEX**	**1L**	**2L**
		
**Thoracic Vertebrae**	141	2.1 ± 1.9	2.53 ± 2.1	3.17 ± 2.1	2.7 ± 2.0	2.11 ± 1.5
**Thoracic disc**	141	1.86 ± 1.7	2.37 ± 1.9	2.63 ± 1.7	2.07 ± 1.5	1.76 ± 1.3
**Lumbar Vertebrae**	120	1.58 ± 1.3	1.8 ± 1.4	1.85 ± 1.5	1.74 ± 1.7	1.39 ± 1.2
**Lumbar Disc**	120	2.52 ± 2.1	3.92 ± 2.5	5.03 ± 2.6	3.26 ± 2.5	1.98 ± 2.0

Abbreviations:

2U-second vertebra/disc above apex

1U-first vertebra/disc above apex

1L-first vertebra/disc below apex

2L-second vertebra/disc below apex

**Figure 4:**
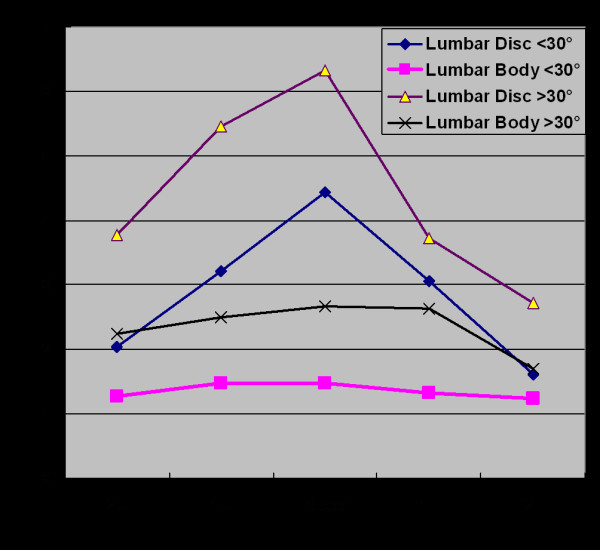
**Wedging in body and disc in lumbar spine**. On the contrary, to thoracic spine, lumbar curve showed an exactly opposite result with greater disc wedging. **X-axis **shows level of wedging measurement. 1 U: 1st upper body or disc. 2 U: 2nd upper body or disc. 1 L: 1st lower body or disc. 2 L: 2nd lower or disc. **Y-axis **shows wedging in of body or disc in degree.

In lumbar spine, the average disc wedging at the apex was 5.03° and it gradually decreased at the ends 2.52° and 1.98° at upper and lower end respectively. Wedging in the body at apex was 1.85° and gradually it decreased at either ends 1.59° and 1.36° at upper and lower end vertebrae respectively, which revealed that there was an overall significant greater disc wedging than vertebral body wedging (p < 0.0001, paired t-test) (Figure 4). The disc wedging was also more profound at the apex. The wedging of the apical disc was found to be significantly more than the disc above and disc below (p < 0.0001, ANOVA test). The wedging was the maximum again towards the curve concavity. However, we could not find significant wedging at the apical vertebral body when compared to level above and below (p = 0.14, ANOVA test). The average values are given in Table 1.

Our result showed that in thoracic spine average wedging at apex at vertebral body was 2.70° and at disc was 2.13° with curve less than 30° while 4.08° and 3.60° at body and disc respectively with curve more than 30°. The apical vertebral body wedging was found to be significantly more in patients having curve more than 30° as compared those having curve less than 30° (p = 0.0002, unpaired t-test). In lumbar spine, the apical wedging at body was 1.47° and at disc were 4.43° with curves less than 30° while 2.67° and 3.72° at body and disc respectively with curves more than 30°. The apical disc wedging was found to be significantly greater (p = 0.0002, unpaired t-test) in the patients with curves more than 30° as compared to those with curves less than 30° (table 2).

**Table 2:** Differential wedging at thoracic and lumbar level according to severity of curve for analysis.

**Description**	**Total no of pt**	**Upper end ± SD**	**2nd upper ± SD**	**1st upper ± SD**	**Apex ± SD**	**1st lower ± SD**	**2nd lower ± SD**	**Lower end ± SD**	**Avg Cobb's ± SD**
**Thoracic Disc All**	141		1.86 ± 1.7	2.37 ± 1.9	2.63 ± 1.7	2.07 ± 1.5	1.76 ± 1.3		27.11 ± 10.6
**Thoracic Body All**	141	1.99 ± 1.8	2.1 ± 1.9	2.53 ± 2.1	3.17 ± 2.1	2.7 ± 2.0	2.11 ± 1.5	1.58 ± 1.2	27.11 ± 10.6
**Lumbar Disc All**	120		2.52 ± 2.1	3.92 ± 2.5	5.03 ± 2.6	3.26 ± 2.5	1.98 ± 2.0		25.13 ± 10.4
**Lumbar Body All**	120	1.59 ± 1.3	1.58 ± 1.3	1.8 ± 1.4	1.85 ± 1.5	1.74 ± 1.7	1.39 ± 1.2	1.36 ± 1.1	25.13 ± 10.4

**Thoracic Disc < 30°**	93		1.7 ± 1.4	1.95 ± 1.5	2.13 ± 1.4	1.67 ± 1.3	1.62 ± 1.2		21.11 ± 5.1
**Thoracic Body < 30°**	93	1.88 ± 1.5	1.82 ± 1.6	1.89 ± 1.6	2.7 ± 1.8	2.18 ± 1.7	1.77 ± 1.4	1.44 ± 1.1	21.11 ± 5.1
**Thoracic Disc > 30°**	48		2.16 ± 2.1	3.17 ± 2.3	3.6 ± 1.7	2.84 ± 1.5	2.01 ± 1.4		38.73 ± 8.0
**Thoracic Body > 30°**	48	2.2 ± 2.2	2.64 ± 2.3	3.78 ± 2.4	4.08 ± 2.4	3.72 ± 2.1	2.75 ± 1.6	1.85 ± 1.4	38.73 ± 8.0

**Lumbar Disc < 30°**	82		2.04 ± 1.9	3.21 ± 2.2	4.43 ± 2.3	3.06 ± 2.2	1.62 ± 1.2		19.38 ± 5.3
**Lumbar Body < 30°**	82	1.39 ± 1.2	1.27 ± 1.0	1.47 ± 1.2	1.47 ± 1.3	1.33 ± 0.9	1.24 ± 1.1	1.2 ± 1.1	19.38 ± 5.3
**Lumbar Disc > 30°**	38		3.78 ± 2.3	5.46 ± 2.6	6.32 ± 2.6	3.72 ± 3.1	2.71 ± 3.0		37.55 ± 8.5
**Lumbar Body > 30°**	38	2.01 ± 1.4	2.24 ± 1.6	2.49 ± 1.5	2.67 ± 1.7	2.63 ± 2.4	1.7 ± 1.3	1.71 ± 1.3	37.55 ± 8.5

**Figure 5:**
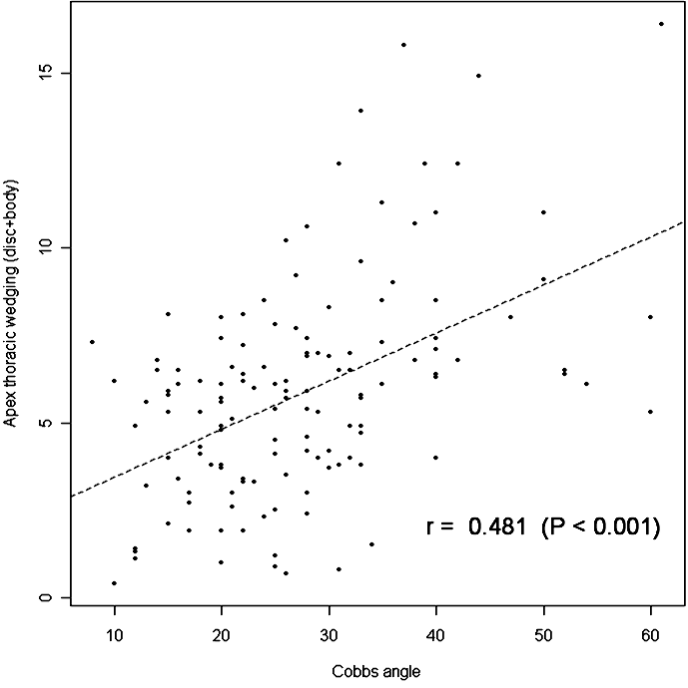
**shows correlation of wedging of apical disc and body according to severity of curve in thoracic curves**. using analysis of covariance. It pointed weak correlation between Cobb angle and wedging in apical disc and body in thoracic curves.

**Figure 6:**
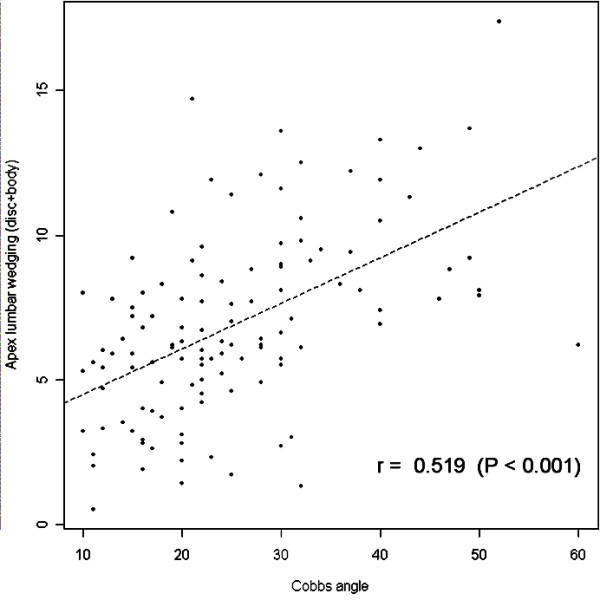
**shows correlation of wedging of apical disc and body according to severity of curve in lumbar curves**. using analysis of covariance. It also indicated weak correlation between Cobb angle and wedging in apical disc and body in lumbar curves.

Comparing the Cobb angle with wedging angle at the apical disc and body using analysis of covariance, it shows that with increasing Cobb angle the wedging angle in the apical disc and body also increases (r = 0.48, p < 0.001 for the apical disc and body in thoracic spine and r = 0.52, p < 0.001 for the apical disc and body in lumbar spine). (Figure 5 and 6) Although these results didn't suggest a strong relationship between Cobb angle and wedging at the apical disc and body, it indicated an existing relationship between two.

Comparing the thoracic vertebral wedging with different grades of rotation, we found that most of the vertebrae had grade 1 rotation and statistically we could not analyze them according to severity of curves.

## Discussion

Our study showed that the wedging of vertebral body and intervertebral disc was consistently present in both the thoracic spine and in lumbar spine. In the thoracic spine, the vertebral body wedging was more distinct as compared to the thoracic disc. However, in the lumbar spine the intervertebral disc wedging was more evident. The progress of the scoliotic curves is commonly thought to as per the Hueter-Volkmann's law [5]. As per this law, epiphyseal growth is inhibited when compressive force act on it, and stimulated when distraction force is applied. Based on this law Roaf [13] has proposed a vicious cycle regarding progression of kyphosis. According to it, a minimal wedging of the vertebrae would produce abnormal compressive force on the vertebral end plate, which would further increase the wedging as per the Hueter-Volkmann's law and thus produce further abnormal forces. Using this principle, scoliotic curves have been reproduced on animal studies, like the one done by Braun et al [2]. He created an idiopathic type of deformity in goats by applying forces across the spine. He also found wedging of the vertebrae similar to that seen in scoliosis in humans. Similarly, Mente et al [8,9] and Stokes et al [10-12], in separate studies on rat-tail models, not only could they create a scoliosis like deformity, but were also able to correct it when the forces were reversed.

In the thoracic spine, the wedging pattern of growth modulation was thus according to the Hueter-Volkmann's law [5]. It was seen more towards the apical region of the curves, gradually decreasing towards the end vertebrae supporting the law. In our study, the maximum wedging was seen in the apical vertebra. This result was consistent with the study of Stokes and Aronsson [11] suggesting disc and vertebral wedging in progressive scoliosis. The current study also shows that load distribution is always concentrated maximum at the apex on the assumption that wedging results from mechanically modulated growth. However, the role of ribs could not be neglected in thoracic spine for scoliosis. Numerous authors [17,18] have reported role of rib deformity as pathogenesis of scoliosis or rib resection for the correction of scoliosis. Xiong and Sevastik [19] did shortening of three concave side ribs in a 7 years old girl with scoliosis and reported 36% correction. However, in a 7 years old child, role of spontaneous correction of the curve should not be forgotten. Sevastik et al [20] has measured rib asymmetry in 10 thoracic idiopathic scoliosis and found that convex rib vertebral angle (RVA) is smaller than concave RVA between T2–T8 and become reverse between T9–T12. Similarly Agostini et al [21] have established relationship between rib hump deformity and vertebral rotation in idiopathic scoliosis. However, in present, we did not measure the difference in RVA between convex and concave side to find out rib asymmetry, which may be a lacuna in this study.

In contrast to the thoracic spine, the lumbar spine showed no significant difference in vertebral body wedging between the apical and other vertebrae; however, it is significant finding that the intervertebral discs follow the same nature of wedging, as it is body in thoracic spine. Actually, in lumbar spine discs are more flexible than thoracic discs; so more stress concentration in disc explain more wedging than the vertebral bodies. According to Stokes and Aronsson [11], among the patients with idiopathic scoliosis who had a thoracic major curve, the wedging at the apex was greater in the vertebrae than in the discs, whereas the opposite was generally found at the apex of the major lumbar and thoracolumbar scoliosis curves. Therefore, the results of their study do not support the hypothesis of Taylor [1] that the wedge deformity begins predominately in the discs and subsequently, with curve progression, the vertebrae become wedged. The division of wedging between vertebrae and discs in thoracic and lumbar curves may be related to the different disc thickness (relative to vertebral height) in these two anatomic regions. Our findings also showed similar results and we therefore agree to their conclusion suggesting that in adolescent idiopathic scoliosis, the wedging in the disc and body will be different according to anatomic region even in same type of curve. Stokes and Morse [22] reported that muscle activation patterns generating spinal loading does not promote curve progression. They did their study in lumbar spine and they thought that scoliosis can adopt different muscle activation and so they did not support muscle role for curve progression. Puustjarvi et al [23] reported in their study that long distance running in digs causes reductions in proteoglycan content of cervical and thoracic discs but increases in lumbar discs. The differences depending on spinal region were attributed to different biomechanical demands, showing the characteristics of mechanical loading may influence disc component. Urban et al [24] noted that solute diffusion into the apical disc (measured by flux of nitrous oxide) was reduced due to abnormal mechanical stress on lumbar disc. They speculated that in scoliosis there is a combination of overload and reduced motion due to disc degeneration that results in curve development in elderly people. In addition, as disc is avascular in nature, reversal of load and stress cannot reverse the disc degeneration and wedging back and probably that is the reason why lumbar curve is difficult to treat with conservative treatment. Moreover, disc wedging is a consistent finding in lumbar scoliosis. This was again more towards the apex of the curve.

Grivas et al [25] has suggested that vertebral body wedging appears later when already Cobb angle increases and in small Cobb angle there is no vertebral wedging. Based on their findings they suggested that when the deformity is initiating, intervertebral disc is found wedged but not vertebra body, due to increased plasticity of disc. Recently they [26] proposed a theoretical model of idiopathic scoliosis pathogenesis describing the role of intervertebral disc in correction of scoliotic curves. They suggested that wedging of the elastic intervertebral disc in the immature scoliotic spine could be reversed by application of corrective forces on it either by bracing or staples, which ultimately create modulation of intervertebral disc composition. However comparing vertebral wedging to disc wedging, our study shows that disc wedging is a far more important component of lumbar scoliosis. Similarly the disc wedging in thoracic spine was far less than the vertebral wedging, stating that vertebral wedging was a much more important component of scoliosis in thoracic spine. The difference could be due to the fact that our study population comprised of established scoliotic patients not those who were initiating the curve. And therefore in thoracic spine we could observe more vertebral wedging than the discs confirming our hypothesis of differential wedging in lumbar and thoracic spine (Table 2). In a similar study, Stokes and Aronsson [10] also showed that in both idiopathic and neuromuscular scoliosis groups of patients, the mean vertebral wedging was more than the disc wedging in the thoracic region; the converse was found in curves in the lumbar and thoracolumbar regions.(greater vertebral body wedging in thoracic spine and disc wedging in lumbar spine.) However, their study contained a small sample of patients as compared to our sample group and the purpose of their study was mainly to document the spinal growth due to vertebral body after the age of ten years. In addition, we also noted more wedging angle in more degree of curve, which again explain that wedging increase with the progression of scoliosis. This finding again confirms the observations of Burwell et al [27] that severe and moderate thoracic idiopathic scoliosis the thoracic hump correlates with Cobb angle and the apical vertebral rotation and lateral asymmetry of the back is the major exterior aspect of scoliosis. Recently Stokes [14,15] said that in a predictive model of the evolution of scoliosis simulating the 'vicious cycle' theory, and using published data, a small lateral curvature of the spine can produce asymmetrical spinal loading that causes asymmetrical growth and a self-perpetuating progressive deformity during skeletal growth. We also agree to these findings and we think our findings of differential wedging pattern in idiopathic scoliosis could be a possible mechanism for the 'vicious cycle' of progression of scoliosis curve.

In present paper we, however, could not study the effect of vertebral rotation according to severity of curve because we have measured the rotations Nash and Moe method which is not in measurement but in grades. Therefore we were not able to analyse statistically with severity of curve as most of the rotations were grade 1. We think that may be the weak point in our study that we could not analyze this ordinal data statistically.

## Conclusion

From this study we can, therefore, conclude that the vertebral wedging is the most important vertebral deformation of the thoracic spine, and the deformation while disc wedging is most deformed structure in the lumbar spine in idiopathic scoliosis and probably, important factor for different disease nature in same group which possibly could produce 'vicious cycle' theory by asymmetric loading for the progression of curve.

## Competing interests

The authors declare that they have no competing interests.

## Authors' contributions

HNM has contributed in conception and design and acquisition of data, analysis and interpretation of data, drafting the manuscript and revising it critically; SWS has contributed in conception and design of data, drafting the manuscript and given the final approval of manuscript; HRS has contributed in acquisition of data, revising the manuscript critically and given the final approval; JHY has contributed in acquisition of data and analysis and interpretation of data; HJK has contributed in acquisition of data and analysis and interpretation of data; and CHM has contributed in drafting the manuscript and designing of data and revising it critically. All authors read and approved the final manuscript.

## Consent

All authors give their consent to publish this study and accompanying images.
